# Structural characterization of free-state and product-state *Mycobacterium tuberculosis* methionyl-tRNA synthetase reveals an induced-fit ligand-recognition mechanism

**DOI:** 10.1107/S2052252518008217

**Published:** 2018-06-22

**Authors:** Wei Wang, Bo Qin, Justyna Aleksandra Wojdyla, Meitian Wang, Xiaopan Gao, Sheng Cui

**Affiliations:** a MOH Key Laboratory of Systems Biology of Pathogens, Institute of Pathogen Biology, Chinese Academy of Medical Science, No. 9 Dong Dan San Tiao, Dong Cheng Qu, Beijing 100730, People’s Republic of China; b Paul Scherrer Institute, Swiss Light Source, CH-5232 Villigen, Switzerland

**Keywords:** *Mycobacterium tuberculosis*, methionyl-tRNA synthetase, crystal structure, induced fit, antituberculosis drugs

## Abstract

*Mycobacterium tuberculosis* (MTB) caused 10.4 million cases of tuberculosis and 1.7 million deaths in 2016. The incidence of multidrug-resistant and extensively drug-resistant MTB is becoming an increasing threat to public health and the development of novel anti-MTB drugs is urgently needed. Methionyl-tRNA synthetase (MetRS) is considered to be a valuable drug target. However, structural characterization of *M. tuberculosis* MetRS (MtMetRS) was lacking for decades, thus hampering drug design. Here, two high-resolution crystal structures of MtMetRS are reported: the free-state structure (apo form; 1.9 Å resolution) and a structure with the intermediate product methionyl-adenylate (Met-AMP) bound (2.4 Å resolution). It was found that free-state MtMetRS adopts a previously unseen conformation that has never been observed in other MetRS homologues. The pockets for methionine and AMP are not formed in free-state MtMetRS, suggesting that it is in a nonproductive conformation. Combining these findings suggests that MtMetRS employs an induced-fit mechanism in ligand binding. By comparison with the structure of human cytosolic MetRS, additional pockets specific to MtMetRS that could be used for anti-MTB drug design were located.

## Abstract 2

Structural characterization of *M. tuberculosis* methionyl-tRNA synthetase provides valuable information for antibacterial drug development.

## 1. Introduction   

*Mycobacterium tuberculosis* (MTB) infection remains a public health challenge owing to its high morbidity and mortality rates. In 2016, the World Health Organization (WHO) reported that 10.4 million people developed tuberculosis and 1.7 million died from the disease (Chetty *et al.*, 2017 ▸). Moreover, drug-resistant MTB infections are becoming an increasing threat (Bastos *et al.*, 2014 ▸; Hong-min & Xiao-Hong, 2015 ▸). Multidrug-resistant TB (MDR-TB) and extensively drug-resistant TB (XDR-TB) infections have been reported worldwide. Drug-resistant TB often compromises the current anti-TB therapy protocols. Therefore, finding novel drug targets and the development of new drugs against MTB are urgently needed.

Aminoacyl-tRNA synthetases (AARSs) play essential roles in protein synthesis. The 20 essential AARSs are housekeeping enzymes that are present in most organisms. Some microorganisms which do not encode AsnRS and/or GlnRS depend on indirect pathways to synthesize a complete set of aminoacyl-tRNAs (Li *et al.*, 2015 ▸). AARSs are considered to be appealing antimicrobial drug targets and therefore have attracted attention in the past two decades (Gadakh & Van Aerschot, 2012 ▸). AARSs catalyze the transfer of amino acids to their cognate tRNAs, yielding aminoacyl-tRNAs, which are subsequently used in protein synthesis (Gadakh & Van Aerschot, 2012 ▸). The charging of tRNA is a two-step reaction. Firstly, AARSs recognize a specific amino acid in the presence of ATP and the amino acid is adenylated, resulting in the intermediate product aminoacyl-AMP and pyrophosphate (PP_i_). Secondly, the amino acid is charged to the acceptor arm of the cognate tRNA and AMP is released (Ibba & Söll, 2000 ▸). AARSs are divided into two major classes, I and II, based on the architecture of the catalytic domain. Class I AARSs are characterized by the presence of two consensus motifs, HIGH and KMSKS, and a catalytic domain exhibiting a canonical Rossmann fold. Class I members bind to the minor groove of the tRNA acceptor stem and transfer the amino acids to the 2′-hydroxy group of the terminal ribose of tRNA. Based on sequence and structural features, class I AARSs can be further divided into subclasses a, b and c (Deniziak & Barciszewski, 2001 ▸; Ibba & Söll, 2000 ▸; Vondenhoff & Van Aerschot, 2011 ▸; Ingvarsson *et al.*, 2009 ▸).

Methionyl-tRNA synthetase (MetRS) is a member of subclass Ia. MetRS is unique among the essential AARSs because it not only charges methionine to tRNA for elongation of translation, but also charges initiator tRNA to initialize translation (Deniziak & Barciszewski, 2001 ▸). Structural studies of MetRSs from various organisms have identified remarkable differences which allow the design of inhibitors that specifically target protein biosynthesis in bacterial pathogens without affecting host-cell activities (Vondenhoff & Van Aerschot, 2011 ▸). MetRS is considered to be one of the most promising drug targets in combatting bacterial pathogens, including MTB.

Inhibitors of MetRS from *Staphylococcus aureus* (Green *et al.*, 2009 ▸; Critchley & Ochsner, 2008 ▸; Critchley *et al.*, 2005 ▸; Ochsner *et al.*, 2007 ▸) and *Clostridium difficile* (Critchley *et al.*, 2009 ▸; Ochsner *et al.*, 2009 ▸) exhibit potent antibacterial activities and are currently in clinical or preclinical trials. However, no inhibitors targeting MtMetRS have been identified to date. A collection of MetRS structures from *Aquifex aeolicus*, *Thermus thermophilus*, *Pyrococcus abyssi*, *Escherichia coli *and *Mycobacterium smegmatis* (Nakanishi *et al.*, 2005 ▸; Sugiura *et al.*, 2000 ▸; Crepin *et al.*, 2004 ▸; Mechulam *et al.*, 1999 ▸; Ingvarsson & Unge, 2010 ▸) have been determined. Very recently, the structure of *M. tuberculosis* MetRS (MtMetRS), which shares similar structural characteristics with MetRS from *M. smegmatis*, was reported in complex with the intermediate methionyl-adenylate (Barros-Álvarez *et al.*, 2018 ▸). A comparative analysis of the unliganded structure (apo form or free-state enzyme; F-state) and structures complexed either with the intermediate product, analogues or inhibitors (denoted the product state; P-state) revealed conformation changes induced by the ligands. Earlier this year, the crystal structure of human cytosolic MetRS was released (PDB entry 5gl7; H. Y. Cho, H. J. Lee & B. S. Kang, unpublished work), providing valuable structural insights into the development of pathogen-specific MetRS inhibitors.

In this study, we successfully purified recombinantly produced MtMetRS. We characterized its enzymatic activity and determined high-resolution crystal structures of F-state MtMetRS and P-state MtMetRS in complex with the intermediate product methionyl-adenylate (Met-AMP). The F-state MtMetRS crystal structure has a nonproductive active-site conformation that has not previously been observed in other MetRS homologues. Binding of the Met-AMP intermediate restores the active-site conformation, indicating that MtMetRS adopts an induced-fit mechanism in ligand binding. Our findings provide an important structural insight that is necessary for the successful design of inhibitors targeting MtMetRS.

## 2. Materials and methods   

### 2.1. Cloning, protein expression and purification   

The cDNA encoding the gene for MtMetRS (GenBank accession No. CP009480.1) was amplified by polymerase chain reaction (PCR) using the genomic DNA of the *M. tuberculosis* H37RV strain as the template. A coding sequence for a 6×His tag was added immediately upstream of the coding sequence for MtMetRS in the upstream primer for the PCR reaction. The PCR product was then inserted between the NcoI and XhoI sites of the pQE-60 vector (Qiagen) to express MtMetRS with double 6×His tags at the N- and C-termini, denoted dh-MtMetRS. Next, we introduced a stop codon at the 3′-end of the coding sequence of MtMetRS in a PCR reaction, yielding a plasmid expressing MtMetRS with a single N-terminal 6×His tag, denoted MtMetRS. Dh-MtMetRS was only used for structure determination of the apo form, while the singly His-tagged protein was used in all other experiments. Plasmids expressing mutants of MtMetRS were prepared using site-directed mutagenesis (KOD-Plus Mutagenesis Kit, TOYOBO) following the manufacturer’s instructions. The sequences of the resulting plasmids were verified by DNA sequencing before transformation into *E. coli* C3016 competent cells (NEB). The bacterial culture was grown in lysogeny broth at 37°C to an OD_600_ of 0.8. The expression of recombinant MtMetRS was induced by adding 1 m*M* isopropyl β-d-1-thiogalactopyranoside (IPTG), and the bacteria were cultured for a further 10 h at 28°C. The cells were then harvested by centrifugation at 4500 rev min^−1^ for 25 min. The cell pellet was resuspended in lysis buffer consisting of 50 m*M* Tris–HCl pH 8.0, 200 m*M* NaCl, 10 m*M* β-mercaptoethanol, 1 m*M* PMSF , 10 m*M* imidazole. The cells were disrupted by ultrasonication on ice. The lysate was clarified by centrifugation at 13 000 rev min^−1^ for 1 h. The supernatant was mixed with 2 ml Ni–NTA resin (Qiagen) and incubated on ice for 1 h to allow protein binding. The resin was washed thoroughly using lysis buffer before elution with ten column volumes of elution buffer containing 350 m*M* imidazole. The eluted protein was finally purified using a Superdex 200 column (GE Healthcare) pre-equilibrated with gel-filtration buffer consisting of 20 m*M* HEPES pH 7.0, 100 m*M* NaCl, 1 m*M* DTT. The purified MtMetRS was finally concentrated to 8 mg ml^−1^ before crystallization trials.

### 2.2. Crystallization and structure determination   

Unliganded MtMetRS was crystallized by mixing 1 µl dh-MtMetRS protein with 1 µl reservoir buffer consisting of 0.1 *M* calcium acetate, 0.1 *M* sodium cacodylate pH 5.9, 16% PEG 8000 in a hanging-drop vapour-diffusion system at 22°C. The MtMetRS–Met-AMP complex was assembled by mixing MtMetRS with a single N-terminal His tag at 8 mg ml^−1^ with 2.5 m*M* ATP, 2.5 m*M* l-methionine, 5 m*M* MgCl_2_, 1 m*M* DTT. The mixture was incubated on ice for 30–60 min prior to crystallization. The MtMetRS–Met-AMP complex was crystallized by mixing equal volumes of sample and reservoir buffer consisting of 0.2 *M* lithium sulfate monohydrate, 19% PEG 3350, bis-tris pH 6.9 in a vapour-diffusion system at 22°C. Crystal cooling was carried out by soaking crystals in reservoir buffer supplemented with 10% ethylene glycol for 30–60 s before flash-cooling in liquid nitrogen. X-ray diffraction experiments were performed on beamline BL18U at Shanghai Synchrotron Radiation Facility (SSRF), Shanghai, People’s Republic of China, and on beamline X06DA at the Swiss Light Source (SLS), Paul Scherrer Institute, Villigen, Switzerland. Complete data sets were collected and processed with the *XDS* package (Kabsch, 2010 ▸). The crystal structure of unliganded MtMetRS was solved by molecular replacement with *Phaser* (McCoy, 2007 ▸) using the *M. smegmatis* MetRS structure (PDB entry 2x1l; Seiradake *et al.*, 2010 ▸) as the search model. The crystal structure of the MtMetRS–Met-AMP complex was solved by molecular replacement using the unliganded MtMetRS structure as the search model. The building of the atomic models was completed manually using *Coot* v.081 (Emsley *et al.*, 2010 ▸) and refinement was performed using *PHENIX* (Adams *et al.*, 2010 ▸). The final models have excellent refinement statistics and stereochemical quality. All figures depicting structures were prepared using *PyMOL* (http://www.pymol.org). The data-collection, structure-refinement and structure-validation statistics are summarized in Table 1 ▸.

### 2.3. ATP–PP_i_ exchange activity assay   

The catalytic activity of MtMetRS was assessed using a methionine-dependent ATP–PP_i_ exchange assay with minor modifications (Jørgensen *et al.*, 2000 ▸; Ghosh *et al.*, 1991 ▸). Briefly, the reaction mixture (50 µl) consisted of 0.025 mCi [γ-^32^P]-ATP, 50 m*M* potassium HEPES pH 7.5, 4 m*M* potassium fluoride, 0.02% gelatine, 10 m*M* magnesium chloride, 80 m*M* potassium chloride, 2 m*M* PP_i_ and 1 µ*M* MetRS. Kinetic constants for methionine were determined in the presence of 2 m*M* ATP using 0.01–5 m*M* methionine and for ATP in the presence of 2 m*M* methionine using 0.1–10 m*M* ATP. The reactions were performed at 25°C. The relative catalytic activities of the mutants were assayed using reaction mixtures consisting of 0.025 mCi [γ-^32^P]-ATP, 50 m*M* potassium HEPES pH 7.5, 4 m*M* potassium fluoride, 0.02% gelatine, 10 m*M* magnesium chloride, 80 m*M* potassium chloride, 2 m*M* PP_i_ and 1 µ*M* MetRS. The mixture was pre-incubated at 37°C. PP_i_ and gelatine were added to the reaction mixture last. Samples (1 µl) were removed from the reaction mixture at different time points within 10 min and spotted onto a cellulose polyethylene­imine TLC plate. The spots were 1.5 cm from the bottom of the plate and were spaced by 1 cm. Following development with 1 *M* potassium dihydrogen phosphate at 25°C in a glass jar, the plates were visualized and quantified using a Typhoon Trio Variable Mode Imager (GE Healthcare).

### 2.4. Fluorescence-based thermal shift (FTS) assay   

The FTS assay is based on the principle that ligand binding increases the thermal stability of proteins (Pantoliano *et al.*, 2001 ▸). The FTS assay was performed on a CFX96 Real-Time PCR Detection System (Maskell *et al.*, 2015 ▸). Each well of a microplate was loaded with 30 µl solution consisting of 2 µ*M* protein, 5× SYPRO Orange (Invitrogen) and 200 µ*M* ligand in 50 m*M* HEPES pH 8, 10 m*M* MgCl_2_, 80 m*M* KCl. Additionally, negative controls consisting of 30 µl of various ligands and 5× SYPRO Orange solution in buffer were dispensed onto the microplate. The plates were sealed with sealing foil (Roche) and incubated at 4°C for 10 min. The FTS assay was performed on a CFX96 Real-Time PCR Detection System (Bio-Rad) running the following protocol: heating from 10 to 85°C with a 30 s hold time every 0.5°C. The fluorescence intensity (excitation/emission at 450–490/560–580 nm) was measured after each cycle. The assays were performed twice for each condition and the melting-temperature (*T*_m_) values for MtMetRS and mutants were determined by analysing the thermal denaturation curves with the *CFX Manager* 3.0 software.

## 3. Results   

### 3.1. Expression and characterization of MtMetRS   

**Figure 1:**
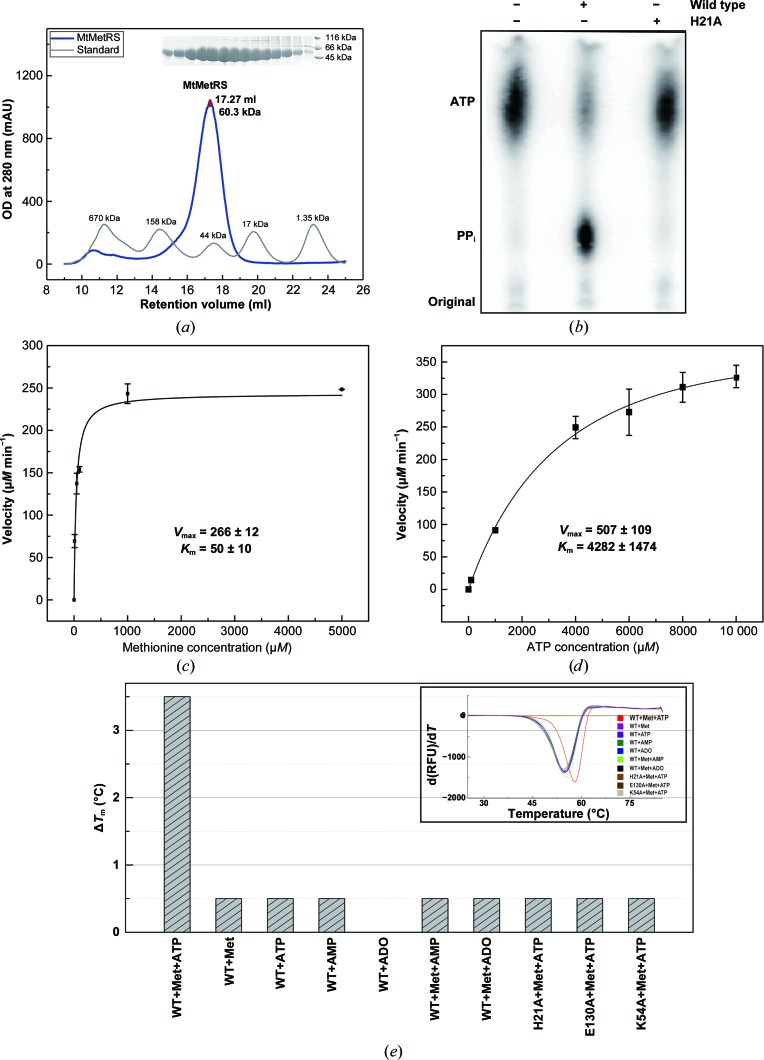
Biochemical characterization of recombinant MtMetRS. (*a*) Size-exclusion chromatography of purified recombinant MtMetRS demonstrating that the enzyme is monomeric in solution. The Superdex 200 300/10 GL column was pre-calibrated with the protein standards thyroglobulin (670 kDa), γ-globulin (158 kDa), ovalbumin (44 kDa), myoglobin (17 kDa) and vitamin B_12_ (1.35 kDa). Upper insert, SDS–PAGE analysis of the eluates. (*b*) ATP–PP_i_ exchange assay showing the catalytic activity of the recombinantly produced MtMetRS. (*c*) The velocity of ATP–PP_i_ exchange is plotted as a function of methionine concentration. The data were fitted to the Michaelis–Menten equation to calculate *V*_max_ and *K*_m_ for methionine. (*d*) The velocity of ATP–PP_i_ exchange is plotted as a function of ATP concentration. The data were fitted to the Michaelis–Menten equation to calculate *V*_max_ and *K*_m_ for ATP. (*e*) Thermal shift analysis of protein–ligand interaction. The *T*_m_ of the F-state MtMetRS was 55°C. The histogram displays the melting-temperature (Δ*T*_m_) shifts of MtMetRS upon incubation with various ligands: Met, ATP, AMP, ADO (adenosine), Met+AMP, Met+ADO and Met-ATP. The thermal shifts of two catalytically inactive mutants, E130A and K54A, were also measured. The concentration of the ligands was 200 µ*M* and the concentration of MtMetRS was 2 µ*M*. In the presence of SYPRO Orange, fluorescence (in relative fluorescence units; RFU) was recorded during heating from 10 to 85°C at a rate of 0.5°C every 30 s. The upper insert indicates the protein melting temperature (*T*_m_).

High-resolution structural characterization of MtMetRS is fundamental to successful targeted structure-based drug design. However, until now the structure of full-length MtMetRS had not been determined owing to difficulties in obtaining soluble protein (Ingvarsson & Unge, 2010 ▸). Therefore, we carried out a systematic optimization of the overexpression conditions, including the selection of an appropriate promoter and bacterial strain, the engineering of suitable affinity tags and optimization of induction, which eventually yielded stable and soluble expression of full-length MtMetRS (see §[Sec sec2]2). We successfully expressed and purified doubly and singly His-tagged MtMetRS. To minimize the impact of the His tag on the activity of the enzyme, we only used the N-terminally singly His-tagged MtMetRS for biochemical characterization. Next, we performed a size-exclusion chromatography experiment. The molecular mass of N-terminally singly tagged MtMetRS calculated from a size-exclusion chromatography (SEC) experiment, 60.3 kDa, closely matched the theoretical mol­ecular mass of 59.3 kDa. This result clearly demonstrates that MtMetRS exists as a monomer in solution (Fig. 1 ▸ *a*), which is consistent with the oligomeric state of its closest homologue *M. smegmatis* MetRS (Ingvarsson & Unge, 2010 ▸). Next, we employed an ATP–PP_i_ exchange assay to evaluate the activity of the recombinantly produced MtMetRS. We found that while the wild-type enzyme exhibited evident ATP–PP_i_ exchange activity, the catalytically inactive H21A mutant displayed an activity similar to that of a control without enzyme (Fig. 1 ▸ *b*). The *k*_cat_ of MtMetRS was calculated as 6 ± 1 s^−1^ and the catalytic efficiency *k*_cat_/*K*_m_ was 0.0015 s^−1^ µ*M* ^−1^ (Figs. 1 ▸ *c* and 1 ▸ *d*). Comparison of the enzyme-kinetic parameters of MtMetRS with those reported for *E. coli* MetRS (EcMetRS; Ghosh *et al.*, 1991 ▸; Table 2 ▸) showed that MtMetRS is less efficient than EcMetRS. To assess whether the N-terminal His tag impacts the activity of the recombinant enzyme, we compared our data with the reported activities of MetRSs from various organisms as summarized in Supplementary Table S3. We found that the *K*_m_^Met^ of MtMetRS was within a reasonable range of reported *K*_m_ values. The *K*_m_^ATP^ of MtMetRS is among the largest reported *K*_m_ values. *E. coli* cells have an average ATP concentration of 1.54 m*M* (Yaginuma *et al.*, 2014 ▸), which is higher than the *K*_m_^ATP^ of EcMetRS. In contrast, the ATP concentration in *M. tuberculosis* is approximately 1.0 m*M* (James *et al.*, 2000 ▸), which is lower than the *K*_m_^ATP^ of MtMetRS. This analysis may offer an explanation of the slow rate of protein synthesis in MTB and the slow growth rate of this bacterium. It is worth noting that the enzymatic activities of human mitochondrial methionyl-tRNA synthetase were characterized using the N-terminally His-tagged enzyme (Green *et al.*, 2009 ▸; Spencer *et al.*, 2004 ▸). Overall, we believe that the presence of the N-terminal His tag does not affect the activity of recombinantly produced MtMetRS.

To guide the co-crystallization of the MtMetRS–ligand complex, we tested the binding affinities of a selection of ligands using a fluorescence-based thermal shift assay. While F-state MtMetRS (the apo form) exhibited a *T*_m_ value of 55°C, we observed different levels of change in *T*_m_ in the presence of various ligands. Among the ligands tested, the largest thermal shift of 3.5°C was observed when methionine, ATP and Mg^2+^ were added to MtMetRS (Fig. 1 ▸ *e*), whereas only negligible thermal shifts (0–0.5°C) were observed for all other ligands. Therefore, a mixture containing MtMetRS, ATP and methionine was selected for co-crystallization. Furthermore, we tested the thermal shifts of two catalytically inactive mutants, K54A and E130A, which were identified based on our structural and mutagenesis studies. Lys54 and Glu130 are involved in the interaction between the catalytic and CP domains and play an important role in maintaining the catalytically productive conformation of the enzyme (see below). We found that despite the presence of ATP and methionine, both mutants showed a negligible thermal shift (0.5°C) that was significantly lower than the wild-type shift (Fig. 1 ▸ *e*).

### 3.2. Structure determination of MtMetRS   

We successfully crystallized both unliganded MtMetRS (denoted F-state MtMetRS) and the enzyme in complex with the intermediate product methionyl-adenylate (Met-AMP; denoted P-state MtMetRS). Dh-MtMetRS with a double His tag was used for the crystallization of the apo-form enzyme, whereas N-terminally His-tagged MtMetRS was used for the crystallization of the Met-AMP-bound enzyme. The crystals of F-state MtMetRS diffracted X-rays to 1.9 Å resolution, belonged to space group *P*1 and contained two copies of MtMetRS in the asymmetric unit. The crystals of the MtMetRS–Met-AMP complex diffracted X-rays to 2.4 Å resolution and belonged to space group *R*3, with a single copy of the enzyme in the asymmetric unit. We located 489 residues in chain *A* and 488 residues in chain *B* out of a total of 519 amino acids in MtMetRS, whereas 504 residues were found in the Met-AMP-bound structure. In particular, there was no electron density for the entire α2 helix between β2 and α3 in the unliganded structure, whereas the α2 region was well ordered in the structure of the MtMetRS–Met-AMP complex. To rule out the possibility that the missing residues in the unliganded structure were a consequence of proteolysis during crystallization, we collected crystals of the unliganded enzyme from the crystallization drops, dissolved the crystals and analyzed the sample by SDS–PAGE. As seen in Supplementary Fig. S1, the protein in the unliganded enzyme crystals migrated similarly to the purified protein. Therefore, we conclude that the residues that are missing in the unliganded MtMetRS structure reflect intrinsic flexibility of the protein and that the binding of Met-AMP stabilizes the catalytic domain. We summarize the data-collection, structure-refinement and validation statistics in Table 1 ▸.

### 3.3. Overall structure of MtMetRS   

**Figure 2:**
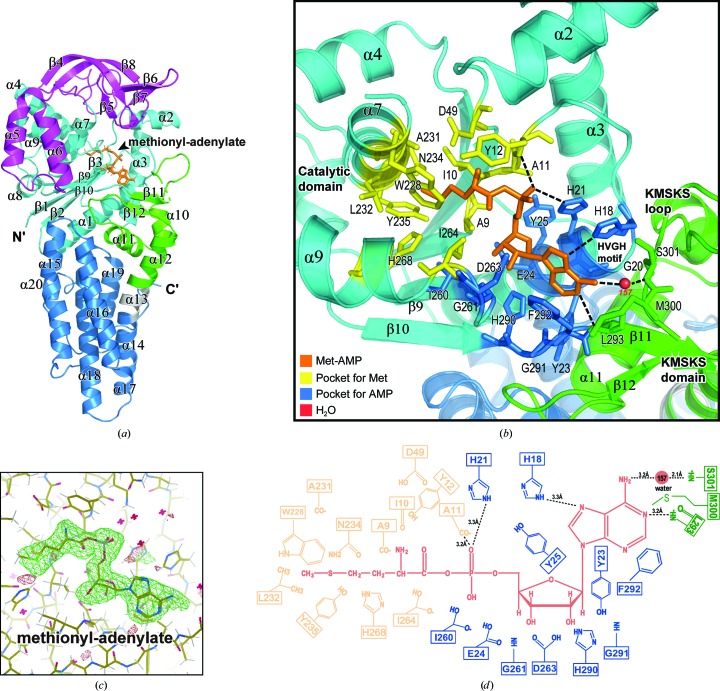
Overall structure of MtMetRS–Met-AMP and the architecture of the active site. (*a*) Ribbon model of MtMetRS. The individual domains are differently coloured, with the catalytic domain in cyan, the CP domain in magenta, the KMSKS domain in green and the anticodon domain in blue. The docking of the intermediate product Met-AMP in the active site is shown as a stick model in orange. The π-helix α13 connecting the KMSKS domain and the anticodon domain is shown in grey. Secondary-structure features of the structure are labelled. (*b*) A magnified view of the MtMetRS structure showing the details of the active site. Residues making up the substrate-binding pockets are shown as stick models and labelled. The methionine pocket is highlighted in yellow and the AMP pocket is highlighted in blue. Met-AMP is shown as a stick model and coloured orange. Ordered waters mediating the interaction between MtMetRS and Met-AMP are shown as red spheres. Hydrogen bonds between MtMetRS and Met-AMP are indicated by dashed lines. (*c*) A magnified view of the structure of the active site with a superimposed polder OMIT map for Met-AMP. The map is contoured at ±3σ with green and red densities. (*d*) A two-dimensional diagram of the active site of MtMetRS occupied by Met-AMP. The colour scheme is the same as that in (*b*). Hydrogen bonds are shown as dashed lines and the bond lengths are indicated.

The overall structure of MtMetRS is similar to those previously reported for MetRSs from various species. The catalytic domain of MtMetRS spanning residues 1–115 (the N-terminal segment) and residues 226–292 (the C-terminal segment) has a typical α/β Rossmann fold. There are five parallel β-strands (β1–β3 and β9–β10) surrounded by seven α-helices (α1–α4 and α7–α9). The connective polypeptide (CP) domain (residues 116–225) is inserted between the N-terminal and C-terminal segments of the catalytic domain. While the α-helix-rich subdomain of the CP domain (α5–α6) tightly binds the α9 helix and the β10 strand of the catalytic domain, the β-rich subdomain of the CP domain (β4–β7) wraps into an arched parallel β-strand covering the top of the active site (Fig. 2 ▸ *a*, Supplementary Fig. S2). The tip of the CP domain is located between β4 and β8, which harbours one ‘knuckle’ but lacks the metal-coordination site. This structural feature classifies MtMetRS into the MetRS1 subfamily (Deniziak & Barciszewski, 2001 ▸). The KMSKS domain (residues 293–350) harbouring the signature _299_KMSKS_303_ sequence (or KMSKS loop) is also known as the stem-contact-fold domain. The KMSKS domain borders the catalytic domain and the anticodon domain. The C-terminal anticodon domain (residues 358–519) is a helix-rich domain comprised of seven antiparallel helices (α14–α20). A small π-helix (α13; residues 351–357) connects the KMSKS domain and the anticodon domain (Supplementary Fig. S2).

We compared the P-state MtMetRS structure with all entries in the Protein Data Bank using the *DALI* server (Holm & Laakso, 2016 ▸; http://ekhidna2.biocenter.helsinki.fi/dali/index.html#tabs-3). The best hit was *M. smegmatis* MetRS (MsMetRS). Structural comparison between MtMetRS and MsMetRS aligned 499 C^α^ atoms and gave a *DALI* *Z*-score of 54.4 and an r.m.s.d. value of 0.8 Å. Comparison of the MtMetRS structure with all other MetRS structures resulted in *DALI* *Z*-scores ranging from 44.2 to 54.4 (Supplementary Table S1).

### 3.4. The active site   

We pre-incubated methionine, ATP and Mg^2+^ with the MtMetRS protein prior to crystallization trials, which allowed the synthesis of an intermediate product in the first step of tRNA charging: methionyl-adenylate (Met-AMP). As expected, we captured a catalytic component complex with Met-AMP fully occupying the active site, providing atomic insight into the recognition of Met-AMP by MtMetRS. The electron-density map (polder OMIT map; Liebschner *et al.*, 2017 ▸) for the intermediate product is very clear, allowing the assignment of all of the Met-AMP atoms (Fig. 2 ▸ *c*, Supplementary Fig. S3). The fit between the experimental electron density and Met-AMP in the final model was assessed using the real-space correlation coefficient (RSCC; 0.95) and the real-space *R* value (RSR; 0.11). These values suggest a suitable fit between the experimental data and the model, and a good agreement between the observed and calculated electron densities for Met-AMP in our structure (Table 1 ▸). Our structure reveals that the active site is located on top of the α1 helix and the C-terminal end of the β-strand plane, and is surrounded by helices α2, α3, α7 and α9. The active site can be divided into a binding pocket for methionine and a binding pocket for AMP. The methionine pocket is a deep cavity built up by 12 highly conserved residues, whereas the AMP pocket is a deep groove formed by 15 conserved residues (Figs. 2 ▸ *b* and 2 ▸ *c*). While methionine and the ribose and adenosine base parts of Met-AMP are deeply buried, the α-phosphate connecting the adenosine and methionine moieties is more exposed to solvent. The O1S atom of the α-phosphate is specifically recognized by multiple hydrogen bonds donated by Ala11 and His21 (Figs. 2 ▸ *b* and 2 ▸ *c*). His18 and His21 belong to the highly conserved HIGH motif (_18_HVGH_21_ in MtMetRS), in which the N^∊2^ atom of the His18 side chain forms a hydrogen bond to the N7 atom of the adenosine base (Figs. 2 ▸ *b* and 2 ▸ *c*). Therefore, the HVGH motif recognizes both the α-phosphate and the adenosine base of Met-AMP. The ribose part of Met-AMP is recognized by several hydrogen-bond interactions. The 2′-hydroxyl group of ribose accepts two hydrogen bonds from the side chain of Asp263, the backbone NH group of Gly261 and the side chain of Ile264. The 3′-hydroxyl group of ribose accepts a hydrogen bond from the side chain of Glu24. The KMSKS loop (located at the tip of the KMSKS domain) forms the distal end of the methionine pocket. The KMSKS loop specifically recognizes the adenosine base of AMP. The adenine ring forms two hydrogen bonds to the carbonyl O atom and backbone NH group of Leu293. The aromatic side chain of Phe292 stabilizes the adenosine base *via* π–π stacking. Additionally, an ordered water molecule (water-157) bridges hydrogen-bond interactions between the N6 atom of the adenosine base and the NH group of Ser301 (Figs. 2 ▸ *b* and 2 ▸ *c*). All contacts between MtMetRS and Met-AMP are summarized in Table 3 ▸.

We compared the architectures of the active sites of MtMetRS and MsMetRS, demonstrating that the residues that mediate the recognition of Met-AMP are highly conserved (Supplementary Fig. S2). Minor differences include Phe292 in MtMetRS, the counterpart of which in MsMetRS is Trp294. Because Phe292 also has an aromatic side chain, it fulfils the same function of stabilizing the adenosine base of AMP *via* π-stacking. A handful of MetRS structures containing Met-AMP (or an analogue) are available in the PDB, including those of *Trypanosoma brucei* MetRS–Met-AMP (TbMetRS; PDB entry 4eg3; Koh *et al.*, 2012 ▸), *Leishmania major* MetRS–Met-AMP–PP_i_ (LmMetRS; PDB entry 3kfl; Larson *et al.*, 2011 ▸), *E. coli* MetRS–methionyl sulfamoyl adenosine (EcMetRS; PDB entry 1pfy; Crepin *et al.*, 2003 ▸) and *A. aeolicus* MetRS–methionyl sulfamoyl adenosine (AaMetRS; PDB entry 2ct8; Nakanishi *et al.*, 2005 ▸). Structural comparison of these MetRS homologues revealed that while the residues recognizing the adenine, phosphate or methionine moieties of Met-AMP remain highly conserved, the residues recognizing the ribose part are more variable (Supplementary Fig. S2).

### 3.5. Extreme changes of the active-site conformation associated with Met-AMP binding   

To analyze the conformational changes associated with Met-AMP binding, we compared the structures of F-state and P-state MtMetRS using the pairwise *DaliLite* server (http://ekhidna.biocenter.helsinki.fi/dali_lite/start). There were two chains (*A* and *B*) in the F-state crystal structure, which are nearly identical. *DALI* pairwise structural alignment between chains *A* and *B* gave a *Z*-score of 58.3 and an r.m.s.d. value of 0.2 Å for 495 (out of a total of 496) aligned residues. Chain *B* was used for further structural analysis because of its lower average *B* factor. To our surprise, the comparison between F-state and P-state MtMerRS resulted in a *DALI* *Z*-score of 46.6 with 479 C^α^ atoms aligned and an r.m.s.d. value of 1.8 Å. This comparison indicates significant differences between the F-state and P-state structures, which are greater than those between MetRS homologues across species (*M. tuberculosis*, *M. smegmatis*, *Brucella melitensis* *etc.*). We further compared the individual domains of F-state and P-state MtMetRS, and found that while the r.m.s.d. between the catalytic domains was 2.1 Å, the r.m.s.d.s between the other domains were less than 1.6 Å (Supplementary Table S2), suggesting that the largest conformational change occurred within the catalytic domain.

**Figure 3:**
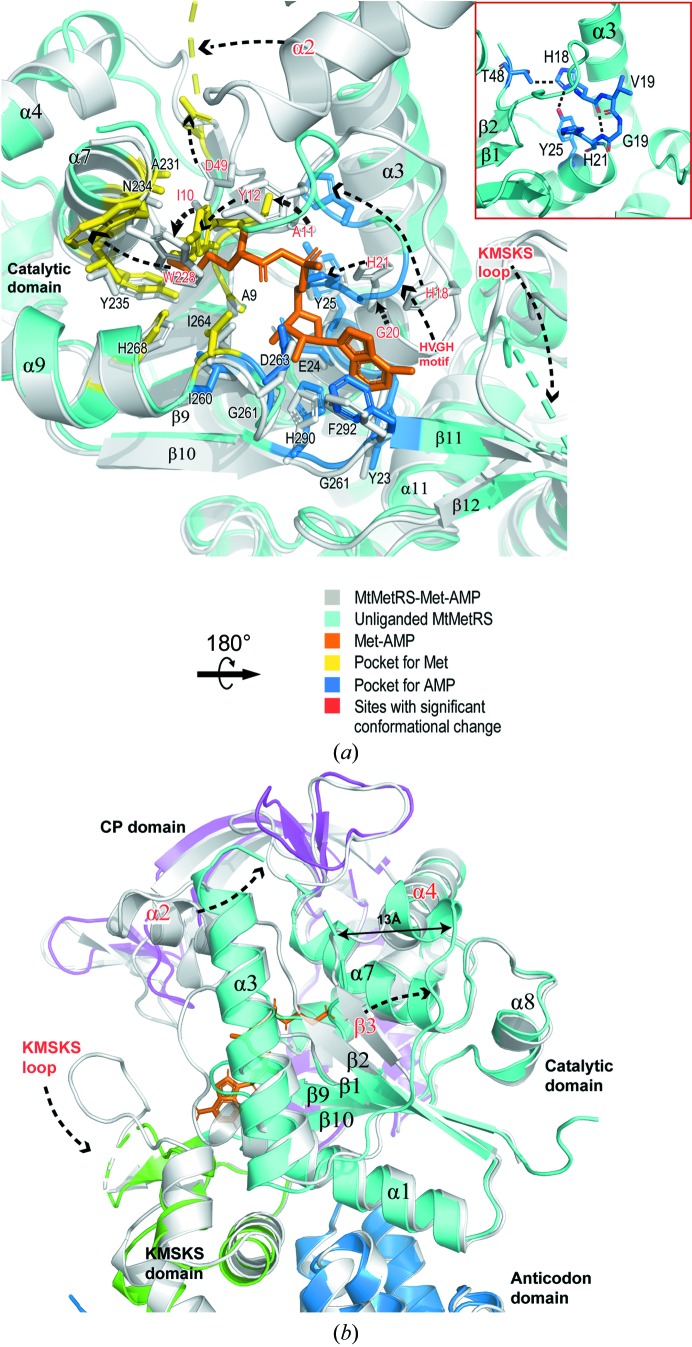
Conformational changes associated with Met-AMP binding. (*a*) The structure of the active site of F-state MtMetRS (cyan) is superimposed with the active site of P-state MtMetRS (grey). The residues that make up the substrate-binding pockets are shown as stick models. The methionine pocket is highlighted in yellow and the AMP pocket is highlighted in blue. Met-AMP bound to the P-state enzyme is shown as a stick model (orange). Large structural rearrangements between these two structures are indicated by the dashed arrows with structural elements labelled in red. The inset shows the unusual β-turn conformation of the HVGH motif in F-state MtMetRS. Secondary-structure elements are labelled. (*b*) View from the opposite side to that in (*a*). Large rearrangements include the KMSKS loop, the α2 and α4 helices and the β3 strand.

We illustrate in Fig. 3 ▸(*a*) that the residues constituting the active site undergo dramatic conformational rearrangements in the absence of the ligand. The nucleotide-binding loop harbouring the conserved HVGH motif does not have a general helical conformation; instead, it adopts a rare type II β-turn conformation (Fig. 3 ▸ *a*). Surprisingly, His18 has moved upwards by 8.2 Å (C^α^ distance) and its side chain is inserted between the parallel β-strands (β1–β3) and the α3 helix. The side chain of His21 tilts towards the α-phosphate of Met-AMP. The KMSKS loop forming the distal end of the AMP pocket was completely missing in the F-state enzyme. Ile10 and Tyr12, which constitute the upper wall of the methionine cavity, push into the methionine pocket (C^α^ displacements of 2.6 and 5.1 Å) and hence the pocket essentially collapses. Moreover, the rearrangement of Tyr12 causes its side chain to clash with the original position of Trp228 (Fig. 3 ▸ *a*), inducing movement of the side chain of Trp228, which constitutes the opposite wall of the cavity, away from the active site.

The large conformational rearrangements of the active site also spread to neighbouring regions (Fig. 3 ▸ *b*). In F-state MtMetRS the entire α2 helix (residues 50–62) connecting β2 and α3 disappeared from the electron-density map, suggesting high flexibility of this region. A polypeptide segment (residues 90–101) located on the opposite side of the active site altered the backbone trace completely (Fig. 3 ▸ *b*). Thr95 from this segment underwent the largest displacement. The C^α^ atom of Thr95 shifted ∼13 Å in the F-state structure with respect to its original position in the P-state structure. As a result, the β3 strand was separated from the β2 strand and the N-terminal part of the α4 helix became distorted.

**Figure 4:**
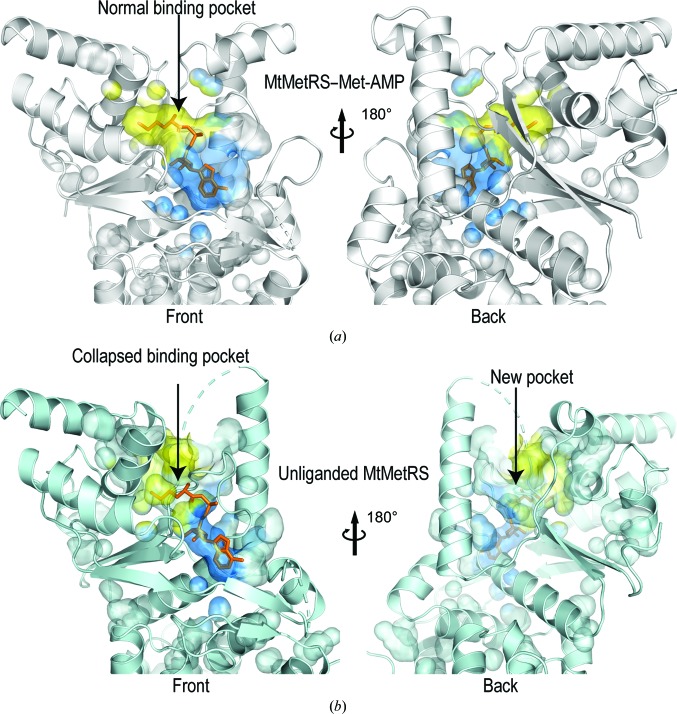
Shrinkage of the active-site cavity in the absence of Met-ATP. (*a*) Ribbon model of P-state MtMetRS (grey) with a semitransparent surface for the cavities and pockets. Left, front view; right, rear view. The methionine pocket is shown in yellow and the AMP pocket in blue. Met-AMP is shown as an orange stick model. (*b*) Ribbon model of F-state MtMetRS (grey) with a semitransparent surface for the cavities and pockets. Left, front view; right, rear view. The methionine pocket is shown in yellow and the AMP pocket is in blue. Met-AMP (orange stick model) is modelled in the active site of the F-state structure by superimposing it with the P-state structure, which demonstrates that the collapsed substrate-binding pockets cannot accommodate Met-AMP.

Collectively, our crystallographic investigations revealed a previously unobserved active site in F-state MtMetRS which appears to be nonproductive. We illustrate in Figs. 4 ▸(*a*) and 4 ▸(*b*) that while the active site is well ordered in P-state MtMetRS, the methionine pocket collapses and the AMP pocket shrinks significantly in the F-state enzyme. We used the *CASTp* software to calculate the volumes of the active sites in both structures (Dundas *et al.*, 2006 ▸). The cavity of the active site has a volume of 2186 Å^3^ in the presence of Met-AMP; in contrast, it reduced to 360 Å^3^ in the absence of the ligand.

### 3.6. Mutagenesis study   

**Figure 5:**
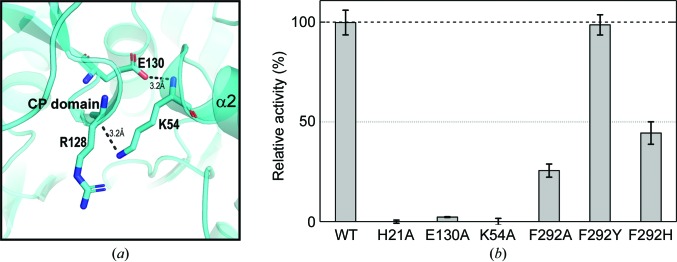
ATP–PP_i_ exchange assay of various MtMetRS mutants. (*a*) Hydrogen bonds between the CP and catalytic domains are shown by dashed lines; the bond lengths are indicated. A histogram presentation of the ATP–PP_i_ exchange activities of a collection of mutants. The catalytic activities of the mutants are expressed as the percentage relative activity compared with wild-type MtMetRS. The result represents triplicate independent measurements with error bars from calculated standard deviations.

To validate our structural findings, we carried out a mutagenesis study. Our structural analyses showed that while the α2 helix was stabilized by the CP domain *via* two hydrogen bonds (from the Glu130 side-chain O^∊2^ atom to Lys53 NH and from the Lys54 side-chain N^ζ^ atom to the carbonyl group of Arg128; Fig. 5 ▸ *a*) in the Met-AMP-bound structure, the corresponding hydrogen bonds were not preserved in the F-state structure and the α2 helix disappeared completely. Using site-directed mutagenesis, we introduced K54A and E130A mutations into MtMetRS to disrupt the hydrogen bond between the CP domain and the α2 helix. We then measured the ATP–PP_i_ exchange activity of these mutants and found that neither the K54A mutant nor the E130A mutant retained activity (Fig. 5 ▸ *b*). The MtMetRS structure shows that Lys54, Glu130 and Arg128 are not in direct contact with Met-AMP; therefore, their essential role in catalysis is more likely to be in the formation of a productive enzyme conformation. This result also suggests that in the absence of ligand the unusual MtMetRS structure observed in the crystals represents a catalytically inactive state.

The conserved aromatic residue Phe292 in MtMetRS is located between the catalytic and KMSKS domains and plays a role in stabilizing the adenosine base of Met-AMP. The corresponding residue in other MetRS homologues varies among phenyl­alanine, tryptophan and tyrosine. We substituted Phe292 with alanine, histidine and tyrosine, respectively. The F292A mutant lost most of the ATP–PP_i_ exchange activity and the F292H mutant lost nearly half of the activity; however, the F292Y mutant showed nearly no loss of activity (Fig. 5 ▸). This result suggests that stabilization of the adenosine base is critical for the formation of Met-AMP.

## 4. Discussion   

In a structural characterization of EcMetRS, Serre and coworkers reported that the binding of methionine induced the rearrangement of several aromatic residues at the active site (Serre *et al.*, 2001 ▸). We observed conformational changes of MtMetRS upon binding by the intermediate product Met-AMP, and the structural rearrangements that occurred in MtMetRS were on a much larger scale. The unusual architecture of the active site of F-state MtMetRS and the large rearrangements of the substrate-binding pockets associated with Met-AMP binding, involving a large displacement of the backbone, alteration of the secondary structure and side-chain rocking, have never been observed in other MetRS homologues. Sequence-conservation analysis of different MetRS homologues showed that the α2 and α3 helices of the catalytic core are less conserved than other parts of the protein. As a result, we found that the hydrogen bonds formed between the α2 and α3 helices and the nucleotide-binding loop in the structures of other MetRS homologues (*E. coli* MetRS, *T. thermophilus* MetRS, *P. abyssi* MetRS and human cytoplasmic MetRS) are absent in the MtMetRS structure. The absence of these hydrogen bonds may be responsible for the high flexibility of the catalytic core of MtMetRS in the absence of ligands. The structure of the MtMetRS–Met-AMP complex demonstrates that the ligand and the CP domain can stabilize the catalytic core and effectively restore the active conformation of the catalytic domain. The unusual F-state structure of MtMetRS observed here reflects the intrinsic flexibility of the catalytic core, which is unique among the characterized MetRSs. This nonproductive conformation probably represents one of the conformations that exist in an ensemble of conformations of MtMetRS.

Crystal packings are nonspecific interactions involving patches on a protein surface with a size that is generally smaller than that of the specific binding interfaces (Carugo & Argos, 1997 ▸). Therefore, crystal packing belongs to the weak protein–protein interactions; it may induce subtle conformational changes to surface loops but rarely alters the folding of a domain core. We analyzed the crystal packing of dh-MtMetRS using *PISA* (http://www.ebi.ac.uk/pdbe/pisa/). The largest packing interface area has a size of 1155 Å^2^ with a Δ^*i*^ *G* of −2.4 kcal mol^−1^, and involved 36 residues of one chain and 39 residues of another. This interaction was not recognized as a specific interaction by the software. We next analyzed the packing interactions in detail, revealing two distinct sites. (i) The N-terminal 6×His tag of one chain occupies a shallow groove located on top of the CP domain of another chain between the α-helix-rich and β-rich sub­domains. The 6×His tag was clearly visible in the electron-density map and is most likely to play a role in lattice formation (Supplementary Fig. S6*a*). Comparing the F-state and P-state MtMetRS structures, which have different space groups and packing interactions, reveals that the structure of the fold of the CP domain remains unchanged, suggesting that the packing force was too weak to affect the conformation. (ii) The catalytic domain of dh-MtMetRS interacts with the CP domain of another chain (Supplementary Fig. S6*b*). The polypeptide segment (between the α3 helix and the α4 helix) exhibiting different conformation in apo and liganded structures is located next to the contact site, but this region is not directly involved in interaction. Another crystal-packing interaction involving the catalytic domain is found between the α3 helix and the α6 helix from the CP domain of a nearby chain (Supplementary Figs. S6*c* and S6*d*). The HVGH motif adopting the rare β-turn conformation in the apo structure is not involved in crystal contacts, suggesting that the observed conformation does not result from crystal-packing artifacts. In the P-state structure, the α2 helix (which is disordered in the apo structure) has a much higher *B* factor (64.8 Å^2^) than the average *B* factor of the entire chain (41.1 Å^2^), which is unusual for a secondary-structural element. The α2 helix of MtMetRS harbours a unique polyalanine segment (residues 58–62) that is followed by a hydrophobic loop between the α2 and α3 helices; thus, this region forms a large hydrophobic patch on the molecular surface. Therefore, our analysis shows that the structure of the α2 region is intrinsically unstable. It is possible that in the absence of the ligand the folding of this region cannot be maintained and it may become disordered, as observed in the F-state structure. Collectively, our analyses demonstrate that it is unlikely that crystal-packing inter­actions resulted in the drastic changes at the active site of the F-state enzyme.

In April 2018, Barros-Álvarez and coworkers reported a crystal structure of MtMetRS complexed with the intermediate product Met-AMP (PDB entry 6ax8; Barros-Álvarez *et al.*, 2018 ▸). It has a lower resolution than PDB entry 5xet; thus, our crystallo­graphic data may provide further structural details. In particular, the clear electron-density map of PDB entry 5xet allowed us to build the intermediate product with high accuracy (Fig. 2 ▸ *c*). The Met-AMP shows a very good fit to the data (RSCC 0.95, RSR 0.11); however, the geometry of the ligand appears to be unusual. In contrast, the Met-AMP in PDB entry 6ax8 had a poorer fit to the data (RSCC 0.84, RSR 0.24) despite exhibiting nearly ideal geometry. To understand this contradiction, we compared the two structures (Supplementary Fig. S7). We found that the aromatic side chain of Phe292 is nearly parallel to the adenine base of Met-AMP in PDB entry 5xet, indicating that Phe292 interacts with the adenine *via* π–π stacking. In contrast, the side chain of Phe292 is almost normal to the adenine plane of Met-AMP in PDB entry 6ax8, suggesting that the π-stacking does not form. This observation suggests that while the adenine base of Met-AMP is recognized by Phe292 in PDB entry 5xet, it is not fully bound in PDB entry 6ax8. Superimposition of the two Met-AMP structures demonstrates that the orientations of their adenine bases account for the largest structural deviation. Therefore, the conformation of Met-AMP observed in PDB entry 5xet might represent an unusual state different from that in PDB entry 6ax8. The conformation of Met-AMP in PDB entry 5xet may represent a metastable state, which coincides with the unusual ligand geometry.

**Figure 6:**
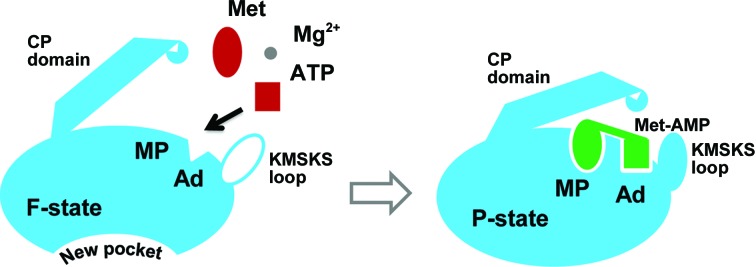
A model of the induced-fit mechanism in ligand binding. Our structural characterization suggests that MtMetRS employs a induced-fit mechanism in ligand binding. In the F-state structure (left) the methionine pocket (MP) does not form and the AMP pocket (Ad) is too narrow to accommodate adenosine. The CP domain is in an open conformation and the KMSKS loop is disordered. In the presence of methionine and ATP, the intermediate product Met-AMP is generated and it accommodates substrate pockets (right). Met-AMP induces the formation of MP and Ad pockets, the architectures of which restore a normal conformation similar to other P-state MtMetRS structures. The CP domain shifts to a closed conformation and the KMSKS loop becomes highly disordered. Owing to the large conformational rearrangements that occur in the F-state structure, F-state MtMetRS exhibits a nonproductive conformation and a new pocket forms on the opposite side to the active site, providing a possible strategy for inhibitor design. Details of the new pocket formed in the F-state structure are shown in Supplementary Fig. S5(*e*).

Koh and coworkers reported eight structures of *T. brucei* MetRS (TbMetRS) complexed with various ligands (Koh *et al.*, 2012 ▸). They revealed a surprising structural plasticity of TbMetRS which allows the enzyme to adopt many different conformations depending on the bound inhibitor. Their analyses suggested that in F-state MetRS the ligand-binding pockets, including the expanded methionine pocket (EMP) and the auxiliary pocket (AP), were pre-formed and open for ligand access. Therefore, they proposed a conformational selection mechanism rather than induced fit. In sharp contrast, we found that F-state MtMetRS is characterized by a completely collapsed methionine pocket, a nonexistent AP (Supplementary Fig. S4) and a severely reduced groove for adenosine. The binding of Met-AMP induced a conformational change in the nucleotide-binding loop and the active site of MtMetRS was restored to a normal P-state conformation that has no significant differences from the P-state conformation observed in other MetRS homologues (Fig. 6 ▸). Combining these analyses suggests that MtMetRS adopts an induced-fit mechanism rather than conformation selection in ligand binding.

Induced-fit and conformation selection are two generally accepted mechanisms for ligand recognition (Vogt & Di Cera, 2012 ▸). The induced-fit mechanism means that the pocket does not pre-exist before ligand binding; thus, the ligand must induce some change in the enzyme structure to form a pocket first. In the case of conformational selection, the pockets are already formed before ligand binding. Different models of ligand recognition have been found in the context of tRNA charging. The bacteria *trans*-editing enzyme ProXp-ala uses a conformation-selection strategy to discriminate Ala-tRNA^Pro^ from Pro-tRNA^Pro^ and thus correct mischarging errors (Danhart *et al.*, 2017 ▸). Datt and Sharma questioned the prevailing induced-fit mechanism for ATP recognition by tyrosyl-tRNA synthetases, which involves conformational changes of the KMSKS loop upon ligand binding (Datt & Sharma, 2014 ▸). Based on a PDB-wide structural analysis, they suggested that an extended conformational selection mechanism is adopted in ATP recognition, rather than an induced-fit mechanism alone.

The induced-fit mechanism employed by MtMetRS may offer an explanation for the slow growth phenomenon of MTB owing to an intrinsic slow protein-synthesis rate. Srivastava and coworkers reported *in vitro* reconstitution of the protein translation system from purified mycobacterial components (Srivastava *et al.*, 2016 ▸). They showed that the *in vitro* protein-synthesis activity of purified *E. coli* components is significantly higher than that of mycobacterial components. Therefore, the slow induced-fit mechanism adopted by mycobacterial MetRS might act as a speed-limiting factor for protein synthesis.

MetRSs play a critical role in both bacteria and humans. Therefore, a prerequisite for a potential drug lead is a high selectivity for the pathogen enzyme over the host enzyme (Ochsner *et al.*, 2007 ▸). There are two human MetRS: a mitochondrial MetRS belonging to the MetRS1 subfamily and a cytosolic MetRS belonging to the MetRS2 subfamily (Gentry *et al.*, 2003 ▸). Structural comparison of human cytosolic MetRS (HcMetRS; PDB entry 5gl7) and MtMetRS gives a *DALI* *Z*-score of 35.8 and an r.m.s.d. value of 1.3 Å with 451 C^α^ atoms aligned. It also shows that HcMetRS and MtMetRS have nearly identical substrate-binding patterns. Interestingly, we found two specific hydrophobic pockets outside the catalytic core of MtMetRS, which might be potential targets for drug design (Supplementary Fig. S5). The inhibitors that bind to either of these pockets might be able to lock MtMetRS in the unbound state and disable MtMetRS activity. This may be a useful target for the design of new drugs targeting *M. tuberculosis*.

In summary, our structural characterization of MtMetRS provides a high-resolution structural framework for drug design. The observed conformational differences between the F-state and P-state MtMetRS structures provide valuable input that is necessary for the successful development of inhibitors specifically targeting *M. tuberculosis*.

## 5. Related literature   

The following references are cited in the Supporting Information for this article: Kalogerakos *et al.* (1980 ▸), Kohda *et al.* (1987 ▸), Nureki *et al.* (1993 ▸), Schmitt *et al.* (1997 ▸) and Schwob *et al.* (1988 ▸).

## Supplementary Material

PDB reference: *Mycobacterium tuberculosis* MetRS, 5xgq

PDB reference: complex with Met-AMP, 5xet

Supplementary Figures and Tables.. DOI: 10.1107/S2052252518008217/jt5026sup1.pdf

## Figures and Tables

**Table 1:** Crystallization conditions, data-collection and refinement statistics and model geometry Values in parentheses are for the outer shell.

	MtMetRS	MtMetRS–Met-AMP
Crystallization conditions	0.1 *M* calcium acetate, 0.1 *M* sodium cacodylate, pH 5.9, 16% PEG 8000	0.2 *M* lithium sulfate monohydrate, 19% PEG 3350, bis-tris pH 6.9
Ligand	—	Met-AMP
Data collection		
Wavelength (Å)	1.0000	0.97776
Beamline	X06DA	BL18U
Synchrotron	SLS	SSRF
Resolution range (Å)	48.69–1.90	37.42–2.38
No. of reflections	287337	104237
No. of unique reflections	158725	49191
〈*I*/σ(*I*)〉	6.49 (0.89)	4.77 (1.15)
Completeness (%)	96.77 (92.13)	99.54 (99.83)
Crystal mosaicity (°)	0.209	0.219
*R*_meas_ (%)	13.1 (108.3)	20.4 (92.1)
Wilson *B* factor (Å^2^)	23.59	31.33
Space group	*P*1	*R*3:*H*
No. of molecules in asymmetric unit	2	1
*a*, *b*, *c* (Å)	49.98, 72.73, 78.38	198.02, 198.02, 39.16
α, β, γ (°)	98.90, 90.05, 98.47	90.00, 90.00, 120.00
Refinement		
*R*_work_	0.20	0.21
*R*_free_ (5% test set)	0.24	0.25
No. of atoms		
Non-H atoms	9079	4205
Protein	7916	3995
Ligand	0	40
Solvent	1163	170
Average *B* factors (Å^2^)		
Overall	30.21	41.06
Protein	28.98	41.17
Ligand	—	45.88
Solvent	38.57	34.01
R.m.s.d.s and stereochemistry		
R.m.s.d., bonds (Å)	0.0033	0.005
R.m.s.d., angles (°)	0.60	0.642
Ramachandran plot, residues (%)		
Favoured region	97.14	97.60
Allowed region	2.86	2.40
Outliers	0	0
Ligand fit with experimental data		
RSCC	—	0.95
RSR	—	0.11
Validation		
*MolProbity* score	1.46, 96th percentile	1.52, 99th percentile
Clashscore, all atoms	5.4, 96th percentile	7.93, 97th percentile
PDB entry	5xgq	5xet

**Table 2:** Pyrophosphate-exchange activity of EcMetRS and MtMetRS

Enzyme	*K*_m_^Met^ (µ*M*)	*K*_m_^ATP^ (µ*M*)	*k*_cat_ † (s^−1^)	*K*_cat_/*K*_m_ ‡ (s^−1^ µ*M* ^−1^)
MtMetRS	50 ± 11	4282 ± 1475	6 ± 1	0.0015
EcMetRS§	21 ± 4	528 ± 91	74 ± 8	0.14

† *k*_cat_ is the average of the values for methionine and ATP.

‡ *K*_m_ is the *K*_m_ for ATP.

§ Ghosh *et al.* (1991 ▸).

**Table 3:** Residues of MtMetRS recognized by Met-AMP (distance cutoff 3.0 Å)

Met-AMP atom	MtMetRS residue	Ordered water
O2S		
P1		
O1S	Ala11, Tyr12, His21	
O1	Ala11, Tyr12	
C9		
C^α^	Trp228	
C^β^	Ala9, Ile10, Ala264, Trp228	
C^γ^	Ile10, Trp228	
S^δ^	Ile10,	
C^∊^	Ile10, Ala231, Leu232, Asn234, Tyr235	
N2	Ile10, Tyr12, Asp49	
O	Ile264	
C5′	Ala11, His21	
C4′	Ala9, Glu24	
C3′	Ile264	
O3′	Glu24, Gly261, Ile264	
C2′		
O2′	Gly261, Asp263, Ile264	
O4′		
C1′	His290	
C4	Ala9, Glu24,	
N3	His290	
C2	His290, Gly291, Phe292	
C8		
N7	His18	
C5		
C6	Gly20	
N1	Gly20, Phe292, Leu293	
N6	Leu293	Water-157
