# Supplementary material for: Structural characterization of free-state and product-state Mycobacterium tuberculosis methionyl-tRNA synthetase reveals an induced-fit ligand-recognition mechanism
Source: IUCrJ. 2018 Jun 22;5(Pt 4):478–90. doi: 10.1107/S2052252518008217 (PMC6038951; doi:10.1107/S2052252518008217)
Supplement: Supplementary file 1 [file m-05-00478-sup1.pdf]

IUCrJ

Volume 5 (2018)

Supporting information for article:

Structural characterization of F-state and P-state *Mycobacterium tuberculosis* methionyl-tRNA synthetase reveals an induced-fit ligand-recognition mechanism

Wei Wang, Bo Qin, Justyna Aleksandra Wojdyla, Meitian Wang, Xiaopan Gao and Sheng Cui

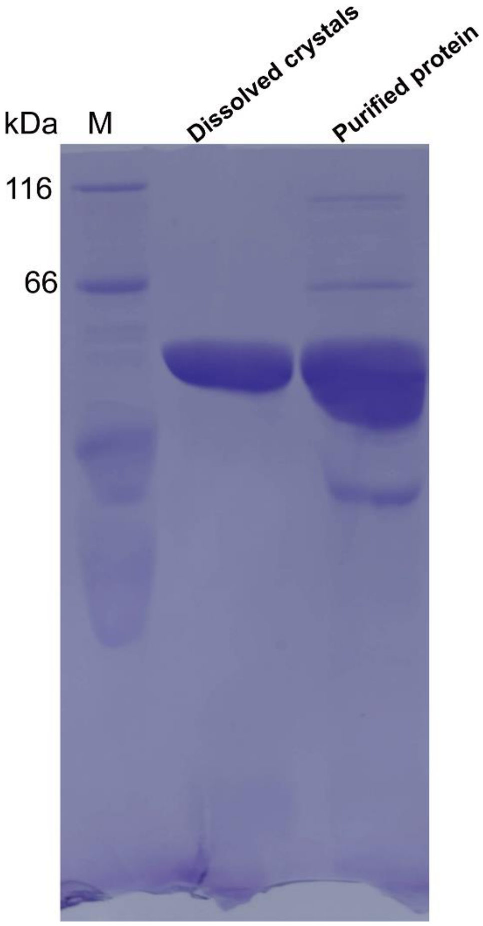

Figure S1 SDS-PAGE of the dissolved unligand MtMetRS crystals. The unligand MtMetRS crystals were washed with reservoir solution substantially and dissolved in loading buffer for SDS-PAGE. The purified protein as a control.

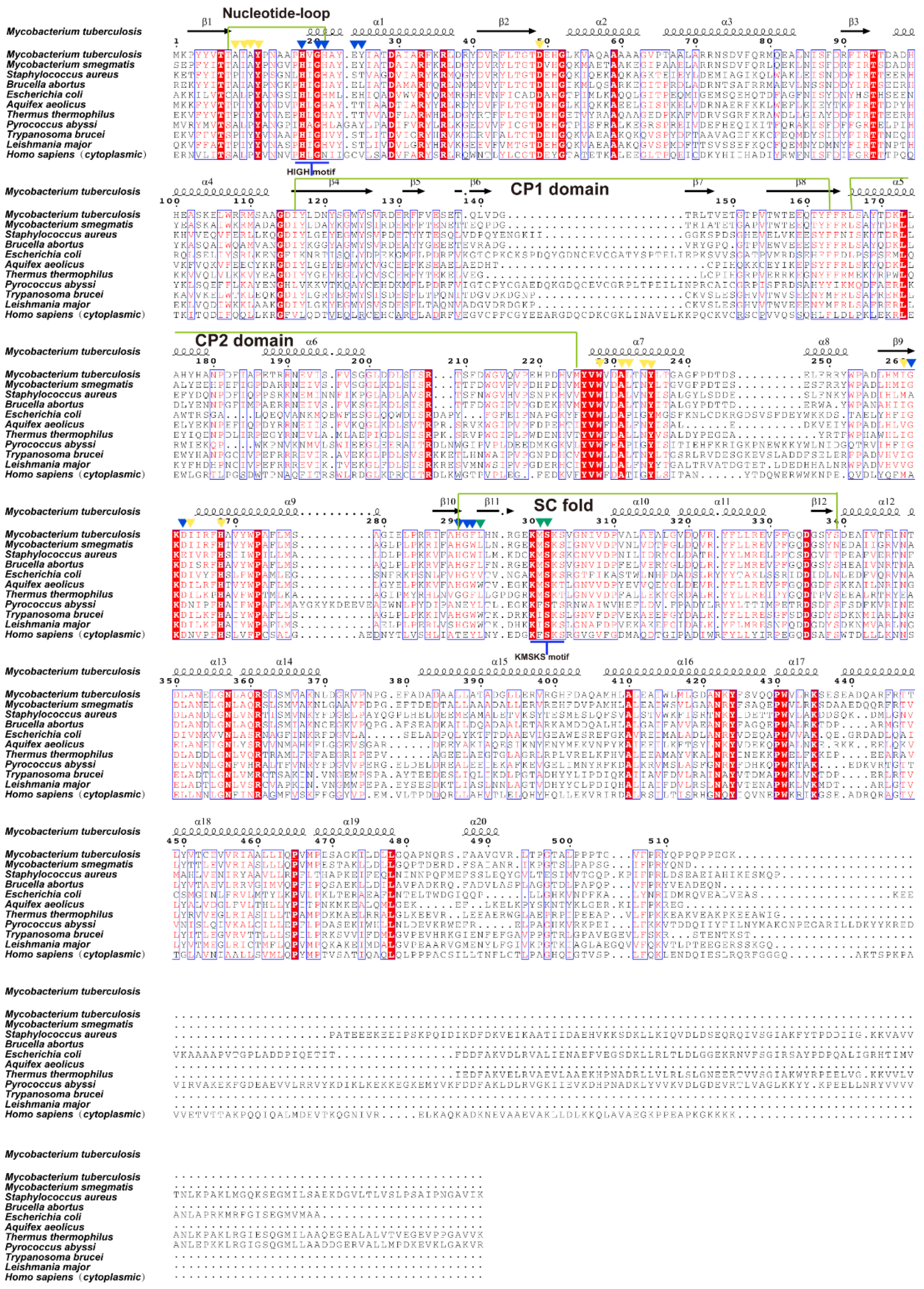

Figure S2 The multiple sequence alignment of MetRS from different species.

Structure-based multiple sequence alignment of MetRS from different species (*M.tuberculosis*, *M.smegmatis*, *E.coli*, *S.aureus*, *T.thermophilus*, *A.aeolicus*, *B.abortus*, *P.abyssi*, *T.brucei* *L.major* and *H.sapiens* (cytoplasm). Conserved residues involved in substrate binding are indicated by triangles that are color coded as in figure 2b. Yellow triangles mark residues

recognizing Met moiety; blue triangles mark residues recognizing ribose moiety; green triangles mark residues recognizing adenine base. The secondary structure elements are represented at the top of the sequences. Conserved motifs are indicated at the bottom of the sequences and domain of catalytic core are indicated at the top of the sequences. The alignment was carried out using the multiple sequence alignment program ClustalW and ESPript 3.0.

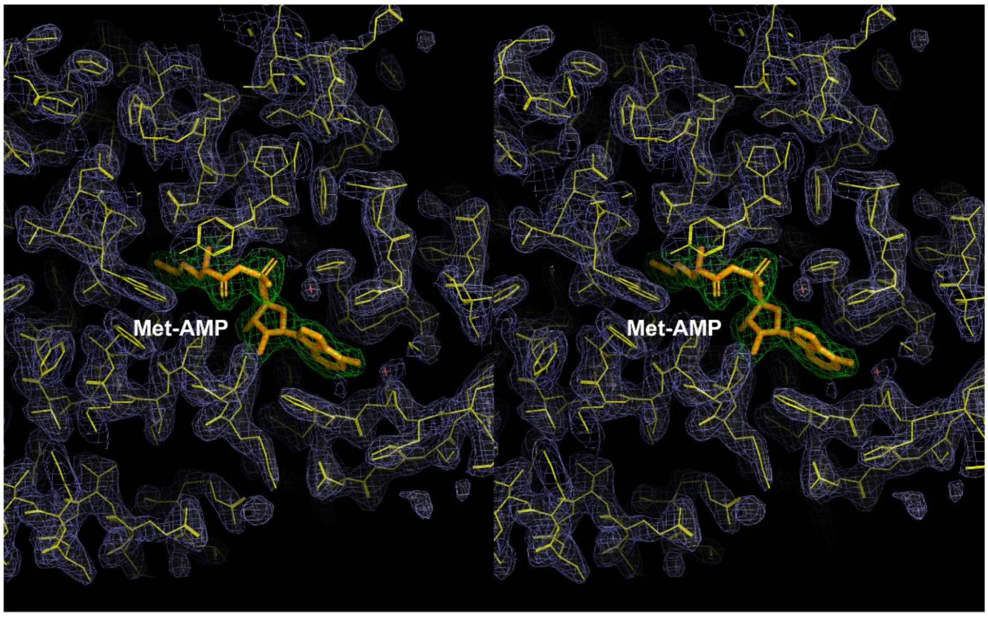

Figure S3 Stereo image of 2Fo−Fc electron-density map for MtMetRS:Met-AMP. Stereodiagram of a ribbon model for MtMetRS:Met-AMP shows the protein(yellow), the ligand Met-AMP(orange) and the 2Fo−Fc electron density map(blue)

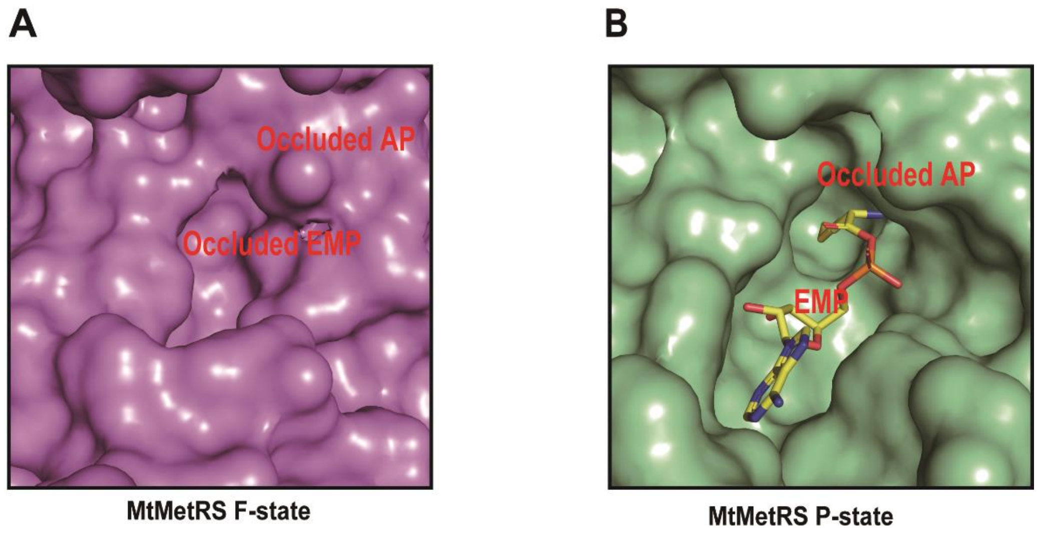

Figure S4 The protein surface of the MtMetRS F state and P state. (A) The protein surface of the MtMetRS F-state (violet),both the EMP and AP are occluded. (B) The protein surface of the MtMetRS P-state (pale green). Met-AMP (yellow) is shown in stick model. the EMP is present but the AP is occluded.

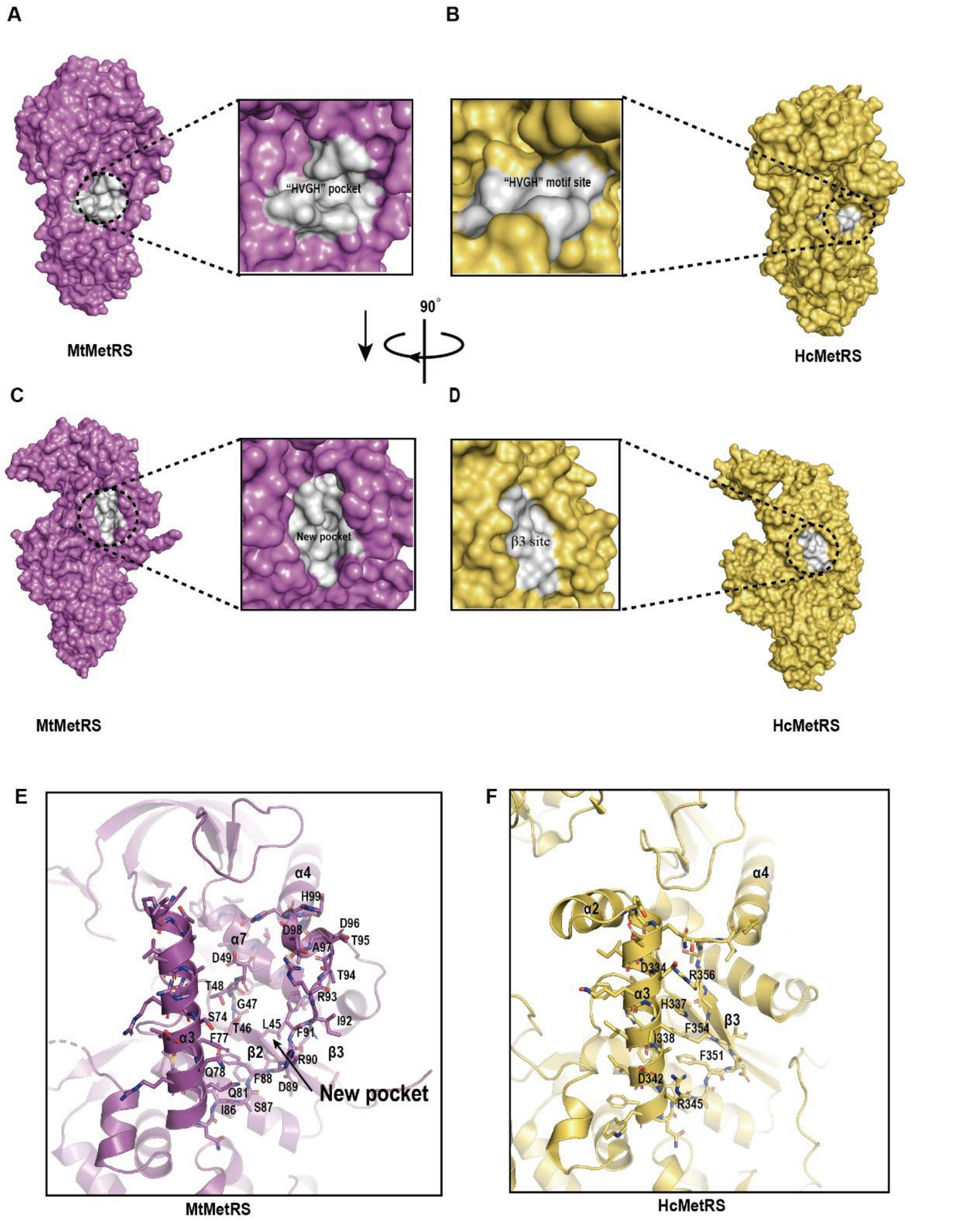

Figure S5 Surface comparison between MtMetRS and HcMetRS. (A) There is a hydrophobic pocket of unligand MtMetRS (violet) (denoted as "HVGH" pocket) between HVGH motif and α10 helix. (B) No hydrophobic pocket was found at the same position of unligand HcMetRS(yellow). (C) There is another hydrophobic pocket (denoted as "New pocket") that exists on the side of unligand MtMetRS (violet). (D) At the same position of unligand HcMetRS(yellow), the hydrophobic pocket does not exist. (E) Show the amino acid

residues that make up New pocket of unligand MtMetRS. (F) Show the amino acid residues of unligand HcMetRS in corresponding with “New pocket”.

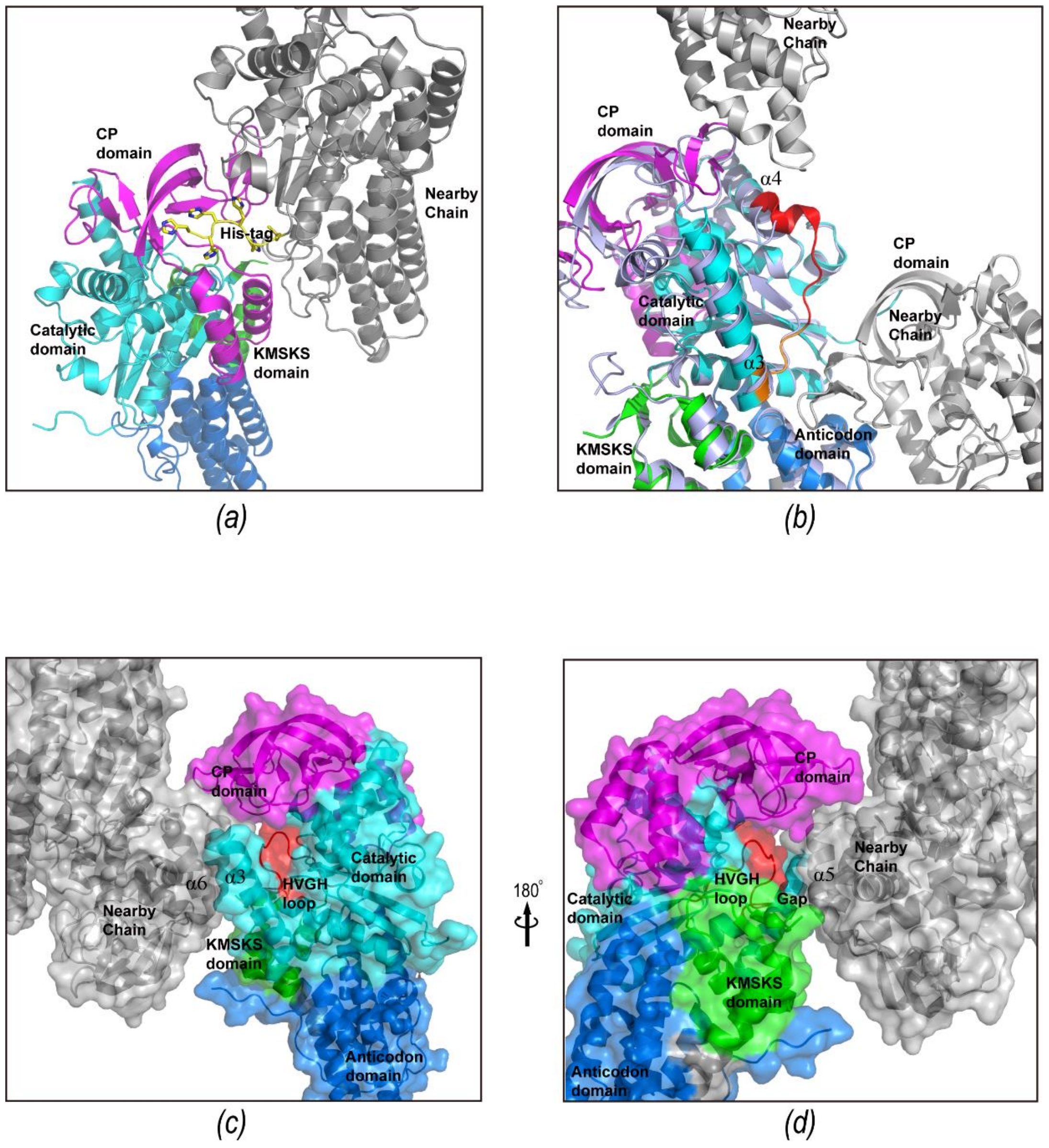

Figure S6 Crystal packing analysis of apo structure of MtMetRS. We analyzed the crystal packing of the dh-MtMetRS crystal structure (apo structure) using software PISA. The model of dh-MtMetRS is colored by domains, the catalytic domain in cyan, the CP domain in magenta, the KMSKS domain in green and the anticodon domain in blue. (a) The N-terminal 6xHis-tag (yellow stick model) of a nearby chain (gray) occupies a shallow groove located on top of the CP domain of dh-MtMetRS, between the α-helix rich and β rich subdomains. The

6xHis-tag was clearly visible in the electron density map and most likely plays a role in lattice formation. (b) The catalytic domain of dh-MtMetRS interacts with the CP domain of a nearby chain. The polypeptide segment (highlighted in red) exhibiting different conformation in the apo structure and the liganded structure (purple) is located next to the contact site (highlighted in orange). (c) Another crystal packing interaction involving the catalytic domain is found between the α3 helix and the α6 helix from the CP domain of a nearby chain (gray). The semi-transparent molecular surface is shown. (d) The view on the opposite side. The HVGH motif located between β1 and α1 (also known as the nucleotide loop, colored in red) adopts a rare β-turn conformation in the apo structure. This region is not in direct contact with the nearby molecule (gray), because there is a gap between the nucleotide loop and the α5 helix. The analysis suggests that the observed conformation is not resulted from the crystal packing artifact.

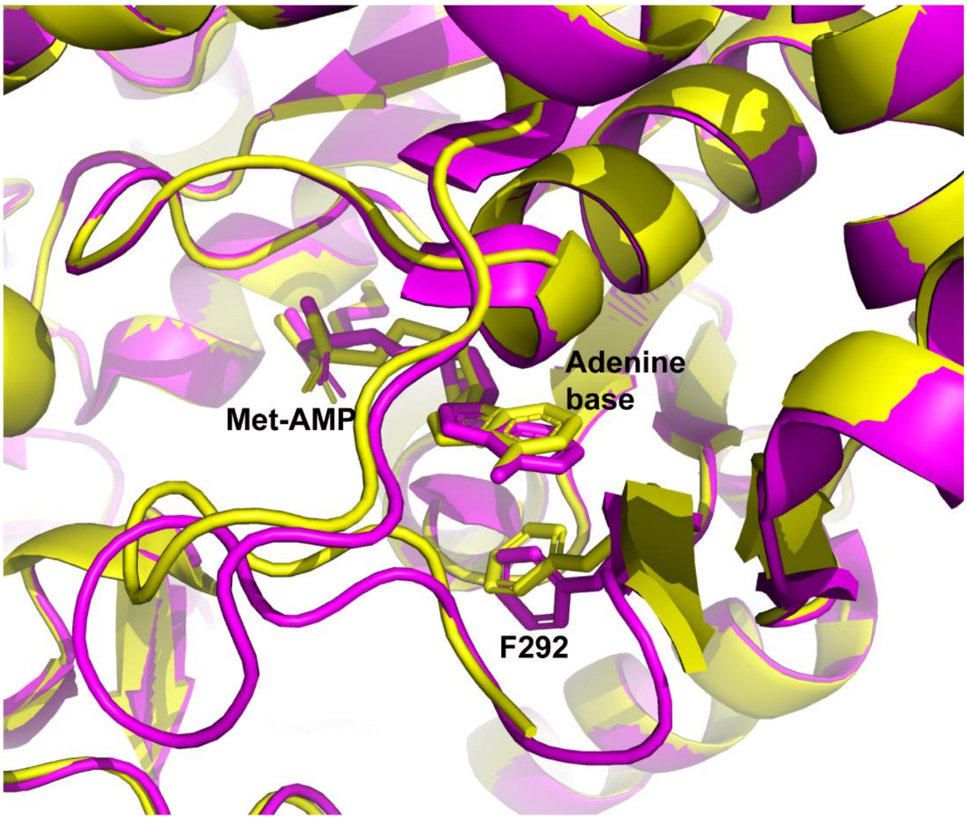

■ **6AX8**
■ **5XET**

Figure S7 Structural comparison of Met-AMP in 6AX8 and 5XET. Two structures of MtMetRS-Met-AMP (PDB entries: 6AX8 magenta and 5XET yellow) are superimposed. The aromatic side chain of F292 is nearly parallel to the adenine base of Met-AMP in 5XET, indicating that F292 interacts with the adenine via a π-π staking. The side chain of F292 is almost normal to the adenine plane Met-AMP in 6AX8, suggesting that the π staking does not form.

Table S1 Structure comparison between P-state MtMetRS and MetRSs from various species

PDB id	Species	Dali Z-score	RMSD(Å)
2x1l	*Mycobacterium smegmatis*	54.4	0.8
4dlp	*Brucella melitensis*	50.7	1.3
5k0s	*Brucella suis*	49.9	2.3
3kfl	*Leishmania major*	46.5	1.6
4eg3	*Trypanosoma brucei*	46.5	1.8
2ct8	*Aquifex aeolicus*	45.1	1.5
2d5b	*Thermus Thermophilus*	45	2.6
4qre	*Staphylococcus aureus*	44.2	2.4

Table S2 Structure comparison between F-state and Met-AMP bound P-state MtMetRS

Domain	Dali Z-score	RMSD(Å)
Catalytic domain	39.4	2.1
CP domain	12.5	1.4
KMSKS domain	8.4	1.6
Anticodon domain	30.7	0.4

Table S3 The enzymatic activities MetRSs from various organisms

Organisms	Substrate K_m(μM)	
	Met	ATP
M. tuberculosis	50	4282
E. coli	21 (Ghosh *et al.*, 1991)	528 (Ghosh *et al.*, 1991)
T. thermophilus	27 (Kohda *et al.*, 1987), 18 (Nureki *et al.*, 1993)	180 [a] (Kohda *et al.*, 1987) 1600[a] (Kohda *et al.*, 1987), 180[b] (Nureki *et al.*, 1993) 1500[b] (Nureki *et al.*, 1993)
H. sapiens mt	18 (Spencer *et al.*, 2004), 20 (Green *et al.*, 2009)	85 (Spencer *et al.*, 2004, Green *et al.*, 2009)
S. aureus	100 (Green *et al.*, 2009)	500 (Green *et al.*, 2009)
S. pneumoniae （MetRS1）	53 (Green *et al.*, 2009)	514 (Green *et al.*, 2009)
H. influenzae	250 (Green *et al.*, 2009)	1200 (Green *et al.*, 2009)
B. stearothermophilus	6 (Kalogerakos *et al.*, 1980), 8 (Schmitt *et al.*, 1997)	12 (Kalogerakos *et al.*, 1980), 11 (Schmitt *et al.*, 1997)
S. cerevisiae mt	6.8 (Schwob *et al.*, 1988)	
S. cerevisiae ct	5.0 (Schwob *et al.*, 1988)	

a. A biphasic profile is observed in the Lineweaver-Burk plot, and two Michaelis constants are obtained.

b. Two K, values for ATP were observed.

mt. mitochondrial

ct. cytoplasmic

Ghosh, G., Brunie, S. & Schulman, L. H. (1991). *The Journal of biological chemistry* **266**, 17136-17141.

Green, L. S., Bullard, J. M., Ribble, W., Dean, F., Ayers, D. F., Ochsner, U. A., Janjic, N. & Jarvis, T. C. (2009). *Antimicrobial agents and chemotherapy* **53**, 86-94.

Kalogerakos, T., Dessen, P., Fayat, G. & Blanquet, S. (1980). *Biochemistry* **19**, 3712-3723.

Kohda, D., Yokoyama, S. & Miyazawa, T. (1987). *The Journal of biological chemistry* **262**, 558-563.

Nureki, O., Kohno, T., Sakamoto, K., Miyazawa, T. & Yokoyama, S. (1993). *The Journal of biological chemistry* **268**, 15368-15373.

Schmitt, E., Panvert, M., Mechulam, Y. & Blanquet, S. (1997). *European journal of biochemistry* **246**, 539-547.

Schwob, E., Sanni, A., Fasiolo, F. & Martin, R. P. (1988). *European journal of biochemistry* **178**, 235-242.

Spencer, A. C., Heck, A., Takeuchi, N., Watanabe, K. & Spremulli, L. L. (2004). *Biochemistry* **43**, 9743-9754.
